# From Simplified Markers to Muscle Function: A Deep Learning Approach for Personalized Cervical Biomechanics Assessment Powered by Massive Musculoskeletal Simulation

**DOI:** 10.3390/s26020752

**Published:** 2026-01-22

**Authors:** Yuanyuan He, Siyu Liu, Miao Li

**Affiliations:** 1 School of Integrated Circuits, Wuhan University, Wuhan 430072, China; 2 School of Power and Mechanical Engineering, Wuhan University, Wuhan 430072, China; 3 School of Microelectronics, Wuhan University, Wuhan 430072, China; 4 School of Robotics, Wuhan University, Wuhan 430072, China

**Keywords:** muscle force estimation, computational musculoskeletal modeling, machine learning, individualized biomechanical analysis, cervical mobility evaluation

## Abstract

Accurate, subject-specific estimation of cervical muscle forces is a critical prerequisite for advancing spinal biomechanics and clinical diagnostics. However, this task remains challenging due to substantial inter-individual anatomical variability and the invasiveness of direct measurement techniques. In this study, we propose a novel data-driven biomechanical framework that addresses these limitations by integrating massive-scale personalized musculoskeletal simulations with an efficient Feedforward Neural Network (FNN) model. We generated an unprecedented dataset comprising one million personalized OpenSim cervical models, systematically varying key anthropometric parameters (neck length, shoulder width, head mass) to robustly capture human morphological diversity. A random subset was selected for inverse dynamics simulations to establish a comprehensive, physics-based training dataset. Subsequently, an FNN was trained to learn a robust, nonlinear mapping from non-invasive kinematic and anthropometric inputs to the forces of 72 cervical muscles. The model’s accuracy was validated on a test set, achieving a coefficient of determination (R^2^) exceeding 0.95 for all 72 muscle forces. This approach effectively transforms a computationally intensive biomechanical problem into a rapid tool. Additionally, the framework incorporates a functional assessment module that evaluates motion deficits by comparing observed head trajectories against a simulated idealized motion envelope. Validation using data from a healthy subject and a patient with restricted mobility demonstrated the framework’s ability to accurately track muscle force trends and precisely identify regions of functional limitations. This methodology offers a scalable and clinically translatable solution for personalized cervical muscle evaluation, supporting targeted rehabilitation and injury risk assessment based on readily obtainable sensor data.

## 1. Introduction

Accurate assessment of cervical spine function necessitates a comprehensive understanding of the forces generated by the neck muscles. Estimating these forces provides crucial insights into cervical biomechanics, facilitates the identification of functional impairments, and informs the development of effective rehabilitation strategies [1,2,3]. However, direct measurement of muscle forces remains challenging due to the complex anatomy of the cervical spine and the limitations inherent in non-invasive measurement techniques [4,5]. These challenges have motivated the development of computational models aimed at more accurately estimating muscle forces.

Several methodologies have been proposed to estimate muscle forces in the cervical spine. Traditional approaches often involve physical models combined with optimization techniques, encompassing both static and dynamic optimization combined with inverse dynamics analysis. Previous studies [6,7] have demonstrated that the integration of inverse dynamics with static optimization yields reasonable approximations of muscle forces and activation patterns. More recently, to improve the adaptability and physiological realism of these models, electromyography (EMG)-driven neuromusculoskeletal models have been introduced. These models integrate neural-driven forward dynamics with static optimization [8,9], enabling personalized calibration of muscle-tendon properties and more accurate estimation of joint torques [10]. Despite their promising advantages, such models often necessitate individualized anatomical data, precise calibration of muscle parameters, and complex fine-tuning of model dynamics. Consequently, they can be resource-intensive in terms of both time and financial costs [11].

To overcome these limitations, recent advancements have introduced data-driven approaches that predict muscle activation and joint forces directly from kinematic data or surface electromyography (sEMG) signals. These methods typically require only a single forward pass through the model, making them particularly suitable for real-time applications. Such data-driven approaches are based on Artificial Intelligence (AI) and its subfields. AI is a broad computer science field focused on developing systems capable of performing tasks that typically require human intelligence. Machine Learning (ML), a principal subfield of AI, enables systems to learn from experience and improve performance without explicit programming, thereby allowing algorithms to identify patterns and generate predictions. Deep Learning (DL), a specialized subset of ML, has gained prominence due to its use of deep neural networks with multiple layers to learn complex data representations, achieving state-of-the-art results in domains such as computer vision and natural language processing. This hierarchical learning capability makes DL particularly well-suited for modeling the intricate relationships between kinematic inputs and muscle forces in biomechanics.

For instance, Rane et al. (2019) [12] employed deep neural networks—a core DL architecture—to map motion space to muscle space, enabling muscle force estimation based exclusively on kinematic data. Burton et al. (2021) [13] utilized various machine learning models—including recurrent neural networks (RNNs), convolutional neural networks (CNNs), both of which are DL architectures, fully connected neural networks, and principal component regression—to estimate joint contact forces and muscle force trends. Despite the advantages of these data-driven approaches, challenges remain in terms of interpretability and generalizability, primarily because these methods often lack explicit physiological or mechanical constraints [14,15]. Moreover, most studies rely on datasets derived from specific populations, thereby limiting the applicability of these models across diverse body types and muscle conditions [16]. This limitation is particularly pronounced in the cervical region, where the complex anatomy and the scarcity of available data impede the development of accurate models for estimating deep muscle forces [17,18].

To address these challenges, we propose a framework that integrates personalized musculoskeletal simulations with deep neural networks. Unlike previous approaches to rely on generic models or limited datasets, our method incorporates subject-specific anatomical parameters—such as neck length, shoulder width, and head mass—to generate millions of individualized models. This anatomically diverse training dataset enables robust, nonlinear mappings from kinematics and individual parameters to the forces of 72 cervical muscles. Importantly, our approach includes a functional assessment module that identifies regions of potential muscle impairment beyond the individual’s accessible range of motion. Validation using concurrent motion capture and electromyography data demonstrates both prediction accuracy and individual adaptability. This approach overcomes prior generalizability limitations by leveraging anatomical diversity, providing a scalable, non-invasive tool for personalized assessment of cervical muscle forces.

The remainder of this paper is organized as follows: Section 2 describes the materials and methods employed for data collection, musculoskeletal modeling, and neural network training. Section 3 presents the performance of our model alongside the results of the validation studies. Section 4 discusses the implications of our findings, study limitations, and comparisons with existing work. Finally, Section 5 concludes the paper and outlines future research directions.

## 2. Methods

**Figure 1:**
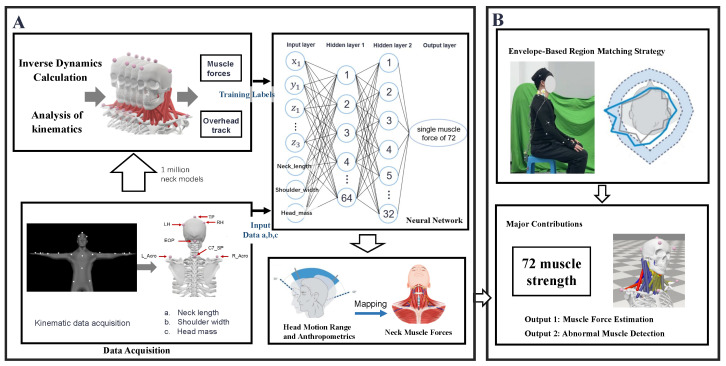
The overall framework of the proposed personalized cervical muscle force estimation method consists of two modules. Module (**A**) constructs a large-scale set of personalized OpenSim models based on individual structural parameters [a. neck length: vertical distance from the seventh cervical vertebra to the external occipital protuberance (C7_SP to EOP); b. shoulder width: linear distance from the left to the right acromion (L_Acro to R_Acro); c. head mass: estimated as 8% of body weight]. Kinematic simulations and inverse dynamics analysis yield head landmark trajectories and muscle forces, which are used to train a neural network for prediction. Module (**B**) maps real-world head motion trajectories into the simulation space and compares them with the ideal motion range to assess muscle function, including muscle force estimation and identification of potential abnormal muscles.

Figure 1 illustrates the overall framework of the proposed method, which integrates large-scale personalized simulation with neural network modeling to estimate neck muscle forces from head motion and individual parameters, as well as to identify potential functional impairments.

### 2.1. Participants

This study involved six healthy subjects (3 males and 3 females; age 25.0 ± 1.6 years; weight 63.5 ± 8.6 kg). None of these individuals had cervical spine lesions or diseases. Additionally, one patient with restricted cervical spine movement due to cervical spine disease was included. The inclusion criteria for the healthy subjects were: (1) no history of cervical spine injury or disease, (2) absence of current neck pain or functional limitations, and (3) ability to perform standardized cervical spine movement tasks. The inclusion criteria for the patient participant were: (1) clinical diagnosis of cervical spine disease, (2) having a clear restriction in cervical spine range of motion, and (3) willingness to participate in the research protocol. All participants underwent screening to ensure they met the inclusion criteria and had no contraindications for motion capture or electromyography (EMG) recording. All participants had signed the informed consent form before participating.

### 2.2. Experimental Protocol and Data Collection

Data collection was performed in two stages: Stage 1 supplied standardized kinematic inputs for Module A in Figure 1, whereas Stage 2 was intended for model validation and functional assessment in Module B.

**Figure 2:**
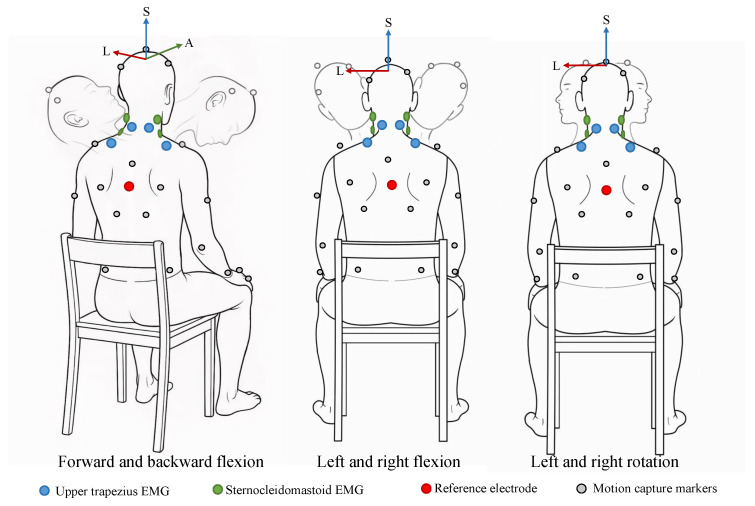
Cervical motion tasks and sensor placement (posterior and lateral views). Blue circles indicate sEMG electrodes placed over the upper trapezius muscles, while green circles denote electrodes over the sternocleidomastoid muscles. The red circle represents the reference electrode. Gray circles correspond to motion capture markers. Axes annotations A, L, and S refer to the anterior, left, and superior directions, respectively.

In Stage 1, six participants performed standardized neck movements—including flexion–extension, lateral bending, axial rotation, and combined motions (Figure 2)—encompassing the full physiological range. These movements were recorded using a 12-camera optical motion capture system (NOKOV, Beijing, China) following the Upper Body marker protocol, which involves multiple markers placed on the head and torso. Specifically, three specific markers were positioned on the head: TP (top of the head), LH (left side of the head), and RH (right side of the head), in accordance with the marker placement guidelines of the XINGYING 4.4-CN protocol (see XINGYING 4.4-CN marker placement (https://docs.nokov.com/xingying/XINGYING4.4-CN accessed on 22 November 2025). These data were sampled at 100 Hz. Joint kinematics were subsequently extracted using the OpenSim neck musculoskeletal model, which represents the cervical spine as a chain of joints extending from the occiput (C0) to the thoracic spine (T1), with degrees of freedom in flexion–extension, lateral bending, and axial rotation. Trajectories from all participants were temporally aligned and interpolated via Dynamic Time Warping (DTW), and the averaged joint angles were used as unified kinematic inputs for simulation.

In Stage 2, one healthy subject and one patient with limited cervical mobility performed the same standardized tasks as in Stage 1, followed by additional spontaneous head movements to capture natural motion patterns. For validation, three markers were placed on the head (TP, LH, and RH) to record three-dimensional head trajectories, which directly define the motion envelope central to our framework. This marker configuration ensures consistency with Stage 1 while also capturing the dynamic range of motion during both standardized and spontaneous movements. Although this setup does not capture all possible cervical rotations, it provides a practical and robust representation of the functional head motion range. Importantly, our approach does not aim to recover exact joint angles or absolute muscle strengths, but rather to identify relative activation patterns and regions of motion limitation.

Motion was recorded using the same optical motion capture system at 100 Hz. Simultaneously, surface electromyography (sEMG) signals were acquired from four superficial neck muscles to validate the neural network predictions and evaluate their physiological plausibility. Detailed specifications regarding the EMG acquisition system, electrode placement, and signal processing procedures are provided in Section 2.3.

### 2.3. EMG Measurement and Processing

Surface EMG signals were simultaneously acquired from four superficial neck muscles: the left and right sternocleidomastoid and the left and right upper trapezius (Figure 2). Data acquisition was performed using a wireless acquisition system (Huoxinwei Technology, Wuxi, China) at a sampling frequency of 1000 Hz, with electrode placement following the SENIAM (Surface Electromyography for the Non-Invasive Assessment of Muscles) guidelines [19]. A reference electrode was positioned on the right acromion process to minimize common-mode noise.

All signals were synchronously processed at a sampling frequency of 1000 Hz. Raw sEMG signals were band-pass filtered (20–450 Hz) using a fourth-order Butterworth filter, full-wave rectified, then low-pass filtered at 10 Hz to obtain the linear envelope [20]. Thereafter, the sEMG data were normalized to each participant’s maximal voluntary contraction (MVC) to enable comparisons across subjects and conditions, while ensuring alignment with the TOH coordinate system for consistent representation.

### 2.4. Personalized OpenSim Model Generation

This study employs the cervical musculoskeletal model developed by Mortensen et al. [21]. To account for anatomical variability, three critical parameters were individualized: neck length, shoulder width, and head mass.

To account for inter-individual variability, three critical morphological parameters were selected for model personalization: neck length, shoulder width, and head mass. These parameters were chosen due to their direct influence on the mechanical loading and force-generating conditions of the cervical musculature.

(a) **Neck length:** An increased cervical segment length extends the lever arm between the head’s center of mass and the cervical joints. For a given head weight and posture, a longer lever arm necessitates greater stabilizing muscle force to maintain equilibrium. In addition, neck length influences the effective path and resting length of muscles spanning the cervical spine, which affects their ability to generate force [22].
(b) **Shoulder width:** A wider shoulder base modifies the lateral attachment sites of several neck muscles. This modification changes their moment arms and fiber orientations, consequently impacting both the efficiency and magnitude of force production, particularly during lateral bending and axial rotation [23].
(c) **Head mass:** An increased head mass elevates the gravitational load that cervical muscles must counteract. Consequently, the muscles are required to generate proportionally greater forces to support static posture and to execute dynamic head movements [24].

These three parameters were therefore identified as the most relevant individual factors for accurately capturing subject-specific variations in neck muscle loading within our simulation framework.

**Figure 3:**
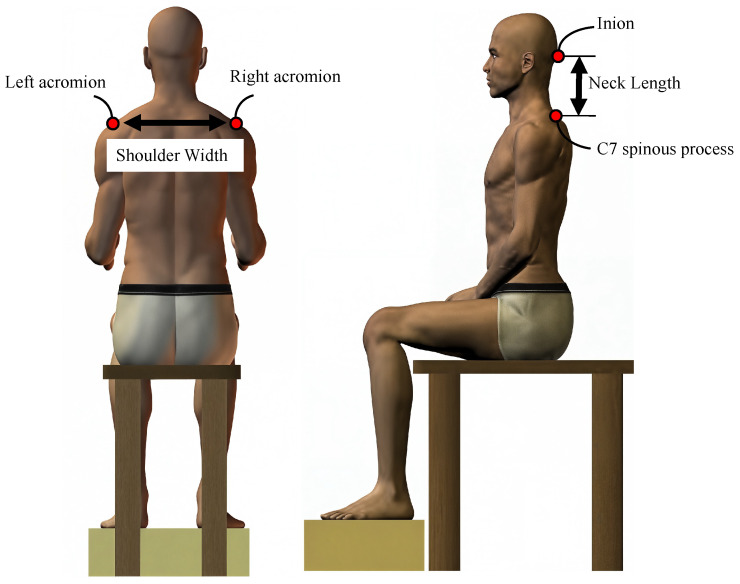
Measurement illustration of neck length and shoulder width. Neck length is defined as the vertical distance from the C7 spinous process to the external occipital protuberance (inion). Shoulder width is the straight-line distance between the left and right acromion. Anthropometric definitions are based on the ANSUR II database [25], which is publicly available at https://www.openlab.psu.edu/ansur2/, accessed on 22 November 2025.

Based on the ANSUR II dataset [25], which includes measurements from 4082 males and 1986 females, the mean vector and covariance matrix of selected anatomical parameters were extracted (Table 1). The covariance matrix captures the variances of each parameter as well as their correlations, thereby ensuring that the generated samples preserve realistic anatomical relationships. A multivariate normal distribution was then used to generate samples reflecting natural anatomical variability. One million plausible models were created, each automatically scaled and adjusted for quality parameters using the OpenSim API. The anthropometric parameters are defined in Figure 3. Head mass was estimated as 8% of body weight [26].

These models established a statistically driven ensemble of musculoskeletal representations that comprehensively encompass human anatomical diversity, ensuring that subsequent representative sampling reflects a wide range of anatomical configurations. This diverse model pool is essential for accurate individual assessments and for effectively representing the full spectrum of human variability in musculoskeletal simulations.

### 2.5. Musculoskeletal Simulation and Muscle Force Estimation

OpenSim 4.4 (https://simtk.org/projects/opensim, accessed on 3 July 2025; official release 27 July 2022) was employed as the primary platform for musculoskeletal modeling and simulation. Utilizing the personalized model library described in Section 2.4, we input the standardized cervical joint trajectories detailed in Section 2.1, which were obtained by averaging time-aligned motion capture data from six healthy subjects. The PointKinematics tool was employed to compute the three-dimensional trajectories of markers in the head coordinate system. These ToH trajectories provided a compact description of overall head motion and were employed to characterize subject-specific ranges of motion.

It is important to note that, although the input joint angles remain consistent, the anatomical parameters of the models vary, leading to changes in muscle length and cervical intervertebral spacing. Consequently, the same joint angle inputs produce different sets of 3D head marker trajectories. These simulations serve as input data for the subsequent neural network, encompassing not only a broad range of achievable head movements but also incorporating individual anatomical variability. This approach ensures that the network learns the nonlinear mapping between kinematics and anatomy, thereby enhancing the model’s generalization capabilities.

To obtain muscle force outputs for each motion frame and to ensure sufficient neural network training samples, 200 models were randomly selected from the personalized musculoskeletal library. This random sampling approach, drawn from the extensive one-million-model library, is essential to mitigate selection bias and to capture a broad spectrum of anatomical variability within the training dataset. The selection of 200 models was a trade-off between computational feasibility and data sufficiency for neural network training. For each model, inverse dynamics (ID) was employed to calculate cervical spine net joint moments based on joint angles, velocities, accelerations, and external loads. The governing rigid-body dynamics of the musculoskeletal system can be expressed as:

(1) $$M\left(q\right)\ddot{q}+C\left(q,\dot{q}\right)\dot{q}+G\left(q\right)=R\left(q\right)f+\tau_{\mathrm{passive}}+J^{⊤}F_{\mathrm{ext}}$$

where q denotes the joint angle vector, $\dot{q}$ and $\ddot{q}$ represent the joint velocities and accelerations, M(q) is the mass matrix, $C(q,\dot{q})$ accounts for Coriolis and centrifugal effects, G(q) is the gravitational torque, R(q) denotes the muscle moment arm matrix, f corresponds to the vector of muscle forces, $\tau_{\mathrm{passive}}$ represents passive joint moments arising from ligaments and connective tissues, and $J^{⊤}F_{\mathrm{ext}}$ corresponds to the generalized forces induced by external loads.

Static optimization (SO) was then subsequently performed to resolve muscle redundancy by distributing the net joint moments among the 72 cervical muscles. To better capture physiological realism and temporal smoothness, several enhancements were incorporated beyond the classical formulation [27,28]. Specifically, a weighted muscle activation term was introduced to emphasize deep stabilizing muscles (e.g., multifidus and longus colli) relative to larger superficial muscles (e.g., sternocleidomastoid and trapezius). Additionally, a metabolic energy term was incorporated to approximate the human tendency to minimize energy expenditure rather than activation alone, and a force rate penalty was included to improve temporal smoothness and prevent abrupt frame-to-frame fluctuations. The combined optimization problem can be formulated as:

(2) $$\min_{f}\sum_{i=1}^{72}w_{i}{\left(\frac{f_{i}}{f_{i}^{\max}}\right)}^{2}+\lambda_{1}\sum_{i=1}^{72}\Phi_{i}\left(f_{i},{\dot{f}}_{i}\right)+\lambda_{2}\sum_{i=1}^{72}{\dot{f}}_{i}^{2}$$

subject to

(3) $$Rf=\tau ,\;0\leq f_{i}\leq f_{i}^{\max},\;f_{j}\approx \kappa f_{k}$$

where $f_{i}$ denotes the force of the *i*-th muscle, $f_{i}^{\max}$ is its maximum isometric force, $w_{i}$ is a weighting factor emphasizing deep or superficial muscles, $\Phi_{i}\left(f_{i},{\dot{f}}_{i}\right)$ represents the metabolic energy expenditure of the *i*-th muscle, ${\dot{f}}_{i}$ is the rate of change of muscle force, $\lambda_{1}$ and $\lambda_{2}$ are weight coefficients for the energy and force rate penalties, and κ constrains co-activation or symmetry between muscles. The weighting factors were set to $w_{\mathrm{deep}}=0.8$ for deep stabilizing muscles and $w_{\sup}=1.2$ for superficial muscles to prioritize deep stabilizers. The penalty coefficients $\lambda_{1}=0.01$ and $\lambda_{2}=0.001$ were chosen to balance metabolic efficiency and temporal smoothness, while κ=1.0±0.05 enforced bilateral symmetry. In this study, κ was selected to be exactly 1.0.

These enhancements improve the physiological fidelity and temporal stability of the predicted muscle forces: weighted activations prevent the underestimation of deep muscles, the energy cost term promotes realistic coordination, the force rate penalty ensures temporal smoothness, and co-activation constraints capture neural control characteristics [29].

This procedure yielded over 8.64 million labeled muscle force samples, which, together with the coordinate positions of the three head markers and individual anatomical parameters, constituted a large-scale dataset for training high-dimensional regression models (the dataset generated in this study is not publicly available but can be shared upon reasonable request from the corresponding author).

### 2.6. Neural Network Training

#### 2.6.1. Data Preprocessing

To prevent imbalance arising from differences in scale and physical units among the input variables, all variables were standardized using Z-score normalization:

(4) $${\hat{x}}_{i}=\frac{x_{i}-\mu_{x}}{\sigma_{x}}$$

where $x_{i}$ denotes the original value, and $\mu_{x}$ and $\sigma_{x}$ represent the mean and standard deviation across all samples. This normalization procedure was applied independently to the ToH coordinates.

The input features, together with muscle forces computed from static optimization, formed a supervised learning dataset. This dataset was first randomly partitioned into a combined training and validation set (80%) and an independent testing set (20%). Subsequently, the combined training and validation set was further split, with 80% used for training and the remaining 20% allocated for validation, which was utilized for hyperparameter tuning and early stopping. This data partitioning strategy is also applied in related studies on gait recognition [30], human activity recognition [31], and treatment outcome evaluation [32].

#### 2.6.2. Neural Network Architecture

A Feedforward Neural Network (FNN) was employed to predict cervical muscle forces, as shown in Figure 1. The network architecture was carefully designed to account for the complexity and nonlinearity inherent in biomechanical data, utilizing a standard four-layer sequential structure comprising one input layer, two hidden layers, and one output layer.

The twelve input features consist of two main components. First, the tri-axial coordinates of three optical motion capture markers placed on the top of the head, totaling nine features, which represent the head’s instantaneous posture in three-dimensional space. Second, three anthropometric parameters are included to account for inter-subject variability, thereby improving the model’s generalizability and predictive accuracy. This 12-dimensional input space ($R^{12}$) provides sufficient information to map observable kinematics onto unobservable muscle forces.

The first hidden layer contains 64 neurons, designed to capture the complex nonlinear relationships among the input features. The second hidden layer, consisting of 32 neurons, further refines and optimizes the high-dimensional feature representations by extracting the most relevant components. To enhance nonlinear modeling capabilities and address the vanishing gradient problem, the Rectified Linear Unit (ReLU) activation function is applied to both hidden layers. The output layer contains a single neuron with a linear activation function, which predicts the muscle force exerted by the corresponding cervical muscle.

To avoid interference arising from biomechanical variability among muscles, an independent modeling approach was employed whereby a distinct network was trained for each of the 72 muscles. Although multi-output networks are possible, the muscle-specific differences in activation, force magnitude, and function favor specialized models. All networks shared the same input structure and predicted frame-wise force for the corresponding muscle.

#### 2.6.3. Evaluation Metrics

Following the completion of neural network training, its performance was evaluated using the following two metrics:

(a) **Coefficient of determination ($R^{2}$):** Quantifies the proportion of variance in the target variable that is explained by the model.
(b) **Normalized Root Mean Square Error (NRMSE):** Quantifies the relative deviation between predicted forces and reference values obtained from static optimization. The RMSE is normalized by the range of the reference data, making the metric comparable across muscles.

The calculation is as follows:

(5) $$R^{2}=1-\frac{\sum_{t=1}^{N}{\left({\hat{y}}_{t}-y_{t}\right)}^{2}}{\sum_{t=1}^{N}{\left(\bar{y}-y_{t}\right)}^{2}}$$

(6) $$\mathrm{NRMSE}=\frac{\sqrt{\frac{1}{N}\sum_{t=1}^{N}{\left({\hat{y}}_{t}-y_{t}\right)}^{2}}}{\max\left(y_{t}\right)-\min\left(y_{t}\right)}$$

where ${\hat{y}}_{t}\in R$ denotes the muscle force predicted by the neural network at time frame *t*, and $y_{t}\in R$ represents the corresponding reference value obtained from the OpenSim static optimization (SO). $\bar{y}$ denotes the mean of all $y_{t}$, and *N* is the total number of time frames. Each value corresponds to the force of a single muscle at a specific frame.

### 2.7. Model Validation: Comparative Analysis Based on sEMG

To evaluate physiological plausibility, the sEMG was employed to compare activation patterns. As sEMG reflects neural drive rather than force—which also depends on length, velocity, and maximal strength—an inverse mapping from predicted force to activation was applied for interpretability. Given the limitations of sEMG, including inter-muscle crosstalk and its restriction to superficial muscles, the validation focused on whether the predicted temporal variations aligned with the general timing and relative modulation of measured the recorded signals, rather than focusing on absolute amplitude values.

For one healthy subject and one patient with cervical dysfunction (Section 2.1), personalized OpenSim models were developed based on captured 3D motion data. Muscle lengths and contraction velocities were estimated via inverse kinematics and muscle analysis. Concurrently, head coordinates and anatomical parameters were input into the trained neural network to predict the time-varying muscle force $F_{\mathrm{muscle}}\left(t\right)$.

The corresponding muscle activation a(t) was subsequently calculated using the Hill-type muscle model:

(7) $$a\left(t\right)=\frac{F_{\mathrm{muscle}}\left(t\right)}{F_{\max}\cdot f_{\mathrm{length}}\left(l\right)\cdot f_{\mathrm{velocity}}\left(v\right)}$$

where $F_{\mathrm{muscle}}\left(t\right)\in R$ denotes the predicted muscle force at time *t*, $F_{\max}\in R$ represents the muscle’s maximal isometric force, $f_{\mathrm{length}}\left(l\right)$ and $f_{\mathrm{velocity}}\left(v\right)$ are normalized force–length and force–velocity multipliers as functions of muscle length *l* and contraction velocity *v*, respectively. The resulting activation a(t)∈[0,1] is a unitless scalar indicating relative muscle excitation.

### 2.8. Exploratory Mapping of Motion Deficits to Muscle Function

To investigate the relationship between observed motion limitations and underlying muscle function, spatial motion envelopes were generated for each subject using the Alpha Shape algorithm [33] applied to the 3D trajectories of the three head markers (TP, LH, RH) obtained from the simulation database. For each marker, a corresponding local motion envelope was created to represent its range of motion. A maximal envelope was then derived from all motion trials for each subject and compared with the simulation envelope using the intersection-over-union (IoU) metric:

(8) $$\mathrm{Similarity}=\frac{V_{\mathrm{intersection}}}{V_{\mathrm{union}}}$$

$V_{\mathrm{intersection}}$ and $V_{\mathrm{union}}$ represent the intersection and union volumes of the two envelopes. To identify the simulation model most closely matching the subject’s motion capacity, the maximum IoU value for each of the three markers was computed. Since TP primarily reflects flexion–extension and lateral bending, and LH and RH primarily capture rotational movement (axial rotation), each marker’s IoU value was treated with equal importance.

The final similarity score for each subject was calculated by summing the maximum IoU values across all three markers. The simulation model with the highest total IoU score was identified as the closest match to the subject’s motion capacity.

Predicted muscle forces corresponding to the reference envelope were obtained by inputting the respective head position and anatomical parameters into the neural network. These predicted forces were then compared qualitatively with the forces predicted from the subject’s actual motion envelope. Specifically, muscle forces that exhibited significant deviations—either consistently elevated or reduced—when comparing the subject’s actual motion envelope to the reference envelope were interpreted as potential contributors to motion restrictions or compensatory actions.

This analysis did not precisely identify the affected muscles because only the coordinates of the head markers were used, and the movement of the intervertebral joints was not recorded. However, it established a preliminary verification framework for linking overall movement defects with muscle-level trends. Future studies that include more comprehensive movement measurement data are required to further refine and validate this method.

### 2.9. Statistical Analysis

To quantitatively evaluate the framework’s performance in distinguishing cervical mobility patterns, a focused statistical analysis was performed on the similarity metrics. Specifically, a paired *t*-test was employed to compare the Intersection over Union (IoU) values of the three primary head markers (TP, RH, and LH) between the patient and the healthy subject. This paired design was selected to account for the inherent spatial correspondence between the markers across different subjects. All statistical computations were conducted using MATLAB 2023a. The statistical significance level was predefined at p<0.05. Given the specific focus on individual functional assessment in this validation stage, this statistical test serves as a rigorous benchmark for validating the framework’s diagnostic sensitivity.

## 3. Results

### 3.1. Neural Networks Predict Performance

**Figure 4:**
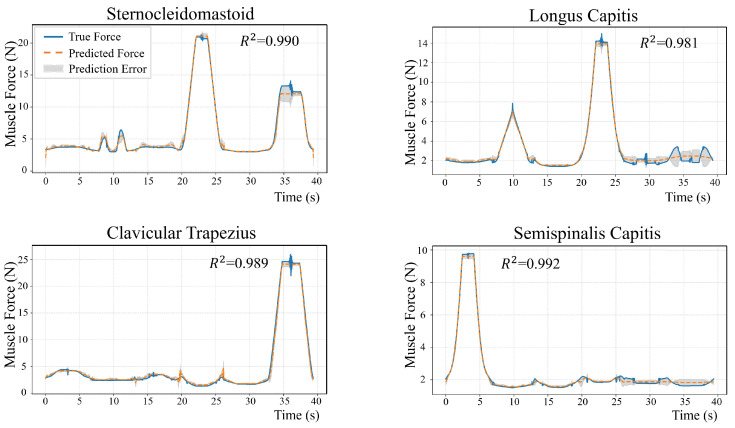
Comparison between predicted muscle forces (orange dashed lines) and OpenSim simulation results (blue solid lines) for four representative neck muscles (sternocleidomastoid, longus capitis, clavicular trapezius, and semispinalis capitis) during a typical motion cycle consisting of flexion–extension, lateral bending, and axial rotation.

A representative full neck motion cycle from the validation set was selected, with the OpenSim simulation serving as the reference for model comparison. Four neck muscles—superficial sternocleidomastoid, trapezius (trap_cl), deep longus capitis (long_cap_sklc4), and semispinalis capitis (semi_cap_sklc5)—were analyzed to evaluate performance across different muscle depths and functional roles. For clarity, only right-side results are shown, as the simulated motions exhibited bilateral symmetry with consistent patterns on both sides. Figure 4 compares the predicted and OpenSim force trajectories, illustrating that the network accurately captured the temporal evolution of muscle forces.

Table 2 presents the RMSE and $R^{2}$ values for each muscle to quantify the prediction performance. The accuracy of the model was evaluated on the test set. For 72 types of muscle strength, the coefficient of determination $R^{2}$ exceeded 0.95. Predictions for superficial muscles (e.g., sternocleidomastoid, trapezius) demonstrated high stability, while deep muscles also demonstrated strong consistency. These results suggest that the proposed network reliably predicts muscle forces across different anatomical depths and functional roles.

### 3.2. Model Validation

**Figure 5:**
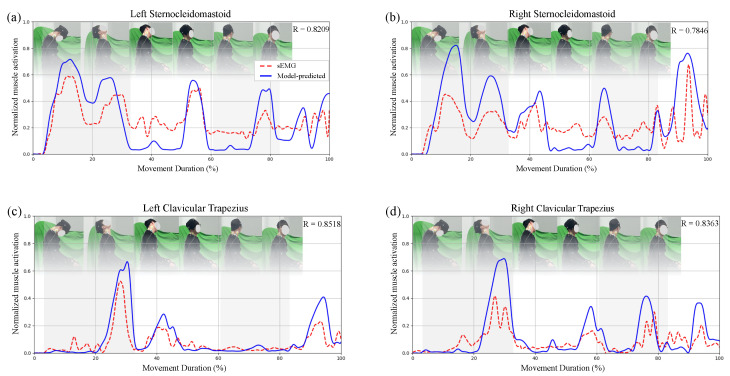
Comparison between normalized sEMG and model-predicted muscle activations in a healthy subject. (**a**–**d**) Results for four superficial neck muscles: (**a**) left sternocleidomastoid, (**b**) right sternocleidomastoid, (**c**) left clavicular trapezius, (**d**) right clavicular trapezius. Red dashed lines represent normalized sEMG signals; blue solid lines show neural network-predicted activations. Gray-shaded areas indicate flexion and axial rotation phases; white areas represent lateral bending and coupled motions. Pearson correlation coefficients (R) are provided in each subplot.

The predicted muscle forces demonstrated strong concordance with sEMG recordings from the bilateral sternocleidomastoid and trapezius muscles, yielding an average Pearson correlation of 0.8234. As illustrated in Figure 5a–d, the model accurately reproduced the expected activation patterns in the healthy subject: the sternocleidomastoid exhibited increased activation during flexion, while the upper trapezius was activated during extension, consistent with their established functional roles [34].

**Figure 6:**
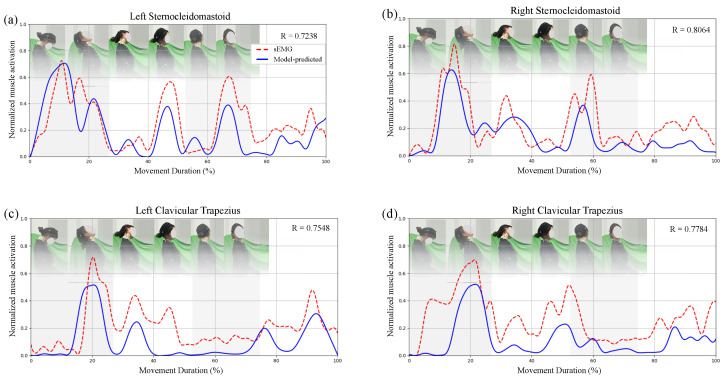
Comparison between normalized sEMG and model-predicted muscle activations in a subject with neck dysfunction. (**a**–**d**) Same four muscles as in Figure 5, presented in the same order. Reduced correlation or altered activation patterns may suggest abnormal neuromuscular control. Red dashed lines: sEMG; blue solid lines: predicted activations. Motion phases and correlation coefficients are denoted as in Figure 5.

Figure 6a–d presents the results for the impaired subject. Significant discrepancies were observed in the right trapezius: sEMG recordings indicated increased activity, which may reflect a compensatory strategy to stabilize the head and shoulder complex. Conversely, the model predicted a reduced force output for this muscle, as the limited range of motion constrained its mechanical demand [35]. Increased activity was also observed in the left trapezius (contralateral side) and the right sternocleidomastoid (ipsilateral side), suggesting that these muscles contributed to maintaining head posture when the primary movers were insufficient. Although sEMG measurements are susceptible to crosstalk—particularly from adjacent superficial neck and shoulder muscles such as the levator scapulae and splenius—the observed activation patterns are consistent with physiologically plausible compensatory mechanisms.

### 3.3. Identification of Impaired Muscles

The intersection over union (IoU) values were calculated for the three head markers (TH, LH, and RH) of both the patient and the healthy subject by comparing their motion trajectories to those in the simulation database. The model with the highest IoU value for each marker was selected for further analysis. Specifically, IoU was calculated by comparing the range of motion of the patient’s actual trajectory with the corresponding range in the simulation database. The model that achieved the highest cumulative IoU value across these three markers was identified as the best match. As presented in Table 3, the average IoU for the patient’s three head markers was 0.7720, while the healthy subject’s average IoU was 0.9275.

**Figure 7:**
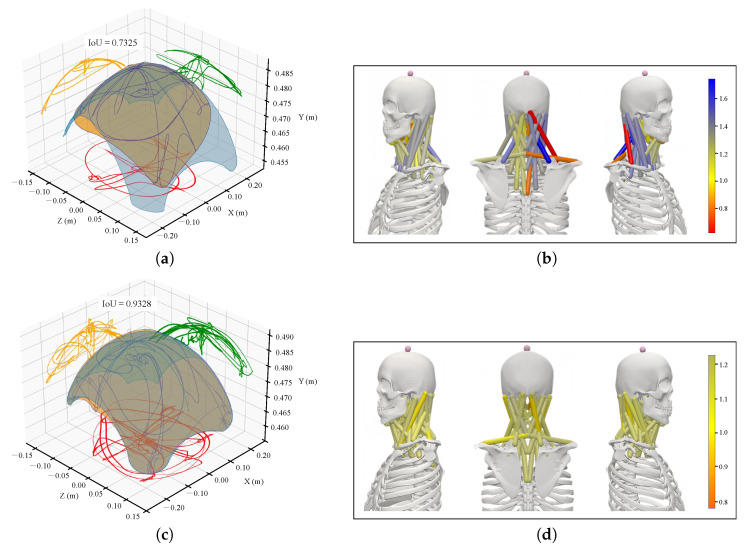
3D visualization of motion-deficit regions and muscle force estimation in a patient with neck dysfunction and a healthy subject. (**a**,**c**) Comparison of subject-specific 3D head trajectory envelope (orange) with the ideal envelope (blue) from the most similar simulated individual, shown for the patient (**a**) and healthy subject (**c**). Projections are shown on XZ (red), XY (orange), and YZ (green) planes. (**b**,**d**) Heatmaps of peak muscle force ratios (actual vs. ideal) mapped onto the OpenSim model from lateral, frontal, and medial views. Red indicates reduced force ratios (possible impairment); blue indicates higher ratios (possible compensation). Ratios near 1 suggest preserved or normal function.

To provide a more intuitive comparison of head motion range, Figure 7a,c present a three-dimensional visual comparison of the head trajectory ranges for the patient and the healthy subject, respectively, with the best-matched models from the simulation database. Notably, the patient exhibited restricted motion in right anterior flexion as well as posterior-lateral flexion.

For the muscle-level analysis, we calculated the ratio of peak muscle forces predicted from the actual trajectory of the subject to those predicted from the ideal trajectory generated by the selected model (Figure 7b). Several deep extensor and rotator muscles on the right side—namely, the longus capitis, semispinalis capitis, and obliquus capitis inferior—exhibited significantly reduced force ratios, indicating underactivation. Components of the right trapezius (trap_acr, trap_cl) also demonstrated diminished force output, suggesting limited torque generation during extension and rotation attributable to restricted range of motion. Conversely, compensatory increases in muscle activation were observed in the right levator scapulae, sternocleidomastoid, and scalene group (force ratios > 1), potentially reflecting the need for stabilization or force redistribution [36].

For the healthy subject, the muscle force ratios (Figure 7d) remained close to 1 across all muscles (ranging from 0.7866 to 1.1732), with no evident signs of compensation. This comparison highlights the model’s ability to detect abnormal force distribution, further supporting its use in identifying potential functional impairments.

### 3.4. Statistical Analysis of Functional Deficits

A statistical comparison was conducted to verify the framework’s efficacy in capturing restricted mobility. The results of the paired *t*-test, as summarized in Table 3, revealed a statistically significant difference in marker IoUs between the patient and the healthy subject (t(2)=−6.81, p=0.029).

This significant reduction in IoU values for the patient group quantitatively substantiates the observed restriction in cervical range of motion compared to the healthy control. Such statistical evidence further demonstrates the framework’s capability to distinguish between normal and impaired cervical function, providing a robust data-driven foundation for personalized biomechanical assessment.

## 4. Discussion

This study presents a novel method for the personalized estimation of neck muscle forces and the non-invasive assessment of functional impairments. The method primarily utilizes individual anthropometric parameters and three-dimensional coordinate data from three relative head markers to predict the forces generated by 72 neck muscles. By utilizing non-invasive data acquisition from the physical world, this approach reduces the reliance on extensive hardware setups. Our framework integrates neural networks with OpenSim simulations to compare and validate the accuracy of neck muscle force predictions. During typical head movements, the model accurately reproduced movement times and overall activation patterns comparable to physiological measurements. The predicted muscle activation was in close agreement with both simulation results and actual surface electromyography (sEMG) recordings. This indicates that combining head top trajectory with anatomical features provides valuable insights into the dynamics of neck muscles.

The primary contribution of this study is the development of an extensive dataset containing one million personalized OpenSim cervical models. By systematically altering three critical anatomical parameters—neck length, shoulder width, and head mass—we effectively simulated the wide range of individual variations commonly observed within populations. This parameterization method ensured both anatomical accuracy and diversity across the generated models.

Another fundamental methodological aspect is the application of spatial motion envelopes for inverse analysis. By comparing the observed head motion in subjects with reference envelopes derived from the simulation database, the framework identifies regions with restricted motion. Based on these underutilized regions, corresponding muscle forces are inferred, thus allowing for the estimation of muscles that may be functionally impaired. While this approach cannot provide precise muscle-specific deficits, it serves as a useful indicator of which muscles might contribute to the restricted motion, including those undetectable by surface electromyography.

In this study, the output data used for model training were obtained through static optimization in OpenSim. It is important to emphasize that the models in OpenSim are primarily based on the assumption of rigid body dynamics, meaning that the components of the body (such as bones and joints) are treated as rigid objects. This assumption neglects the slight deformations that occur in cervical tissues during actual movement, which may influence the accuracy of model training. However, previous studies have shown that even with this simplification, data generated using OpenSim remain reliable [37].

Although optical motion capture systems provide high spatial and temporal resolution for tracking head motion, they do have limitations. These systems are sensitive to variations in illumination, which can affect marker detection, and marker occlusion during certain movements can lead to missing or interpolated data [30,31]. Additionally, the portability of optical systems is restricted by the need for a fixed laboratory setup with calibrated camera arrays, limiting their use in clinical or field settings [32]. Despite these challenges, the optical system was chosen for its superior accuracy and temporal resolution, which are crucial for capturing the detailed kinematic data needed for training the neural network model.

The current validation sample size is relatively small (comprising one healthy subject and one patient), and the subjects are relatively young adults. This limitation may not adequately reflect the variability observed in older populations or those with pathological conditions. Structural degeneration and age-related neuromuscular adaptations could potentially affect neck muscle behavior. Future work should aim to expand the clinical cohort, particularly by including more patients with various types of cervical disorders and individuals across different age groups, in order to comprehensively assess the robustness and clinical diagnostic value of the model.

Future work will enhance the personalized model by integrating muscle morphology data obtained from advanced imaging techniques and expanding the simulation database to encompass a wider range of ages, body types, and clinical conditions. These enhancements are expected to improve anatomical realism and generalizability, thereby supporting applications in wearable devices and clinical systems. We also aim to explore functional connectivity analysis using predicted muscle activation patterns and sEMG data to construct cervical muscle networks. This will enable extraction of graph-theory indices like degree centrality and clustering coefficient, offering insights into muscle synergy and control mechanisms [38,39]. Finally, we will assess the clinical relevance of these metrics for evaluating motor control impairments and rehabilitation outcomes in cervical disorder patients, inspired by studies on perilaryngeal-cranial muscle coherence in vocal tasks [40].

## 5. Conclusions

This study presents a personalized and non-invasive framework for estimating cervical muscle forces by integrating large-scale musculoskeletal simulations with neural network-based predictions. A principal methodological contribution is the reverse analysis of the three-dimensional head movement range envelope, which allows identification of regions with limited movement and facilitates inference of muscles that may be functionally constrained, including those inaccessible through surface EMG.

The framework was evaluated using motion capture and sEMG data, demonstrating a reasonable concordance between predicted muscle force patterns and experimental observations. By incorporating individual anatomical variations and observable head motion trajectories, this approach provides a non-invasive strategy for assessing cervical muscle function and establishes a foundation for quantitative, individualized evaluations in rehabilitation, wearable technologies, and clinical applications. This framework may further support the development of future clinical assessment tools and assistive robotics applications.

## Figures and Tables

**Table 1:** Mean vector and covariance matrix of anthropometric parameters derived from the ANSUR II dataset.

	Head Weight (kg)	Biacromial Breadth (cm)	Neck Length (cm)
Mean	6.377	39.920	10.730
Head weight	1.568	2.678	−0.001
Biacromial breadth	2.678	9.143	0.378
Neck length	−0.001	0.378	1.546

Note: The top row presents the mean values for each parameter, while the lower triangle shows the covariance matrix.

**Table 2:** Performance metrics of neural network predictions for different neck muscles.

Model Name	Muscle Full Name	NRMSE	R^2^	Model Name	NRMSE	R^2^
digastric_post	Posterior Digastric	0.058	0.968	digastric_post_L	0.051	0.982
digastric_ant	Anterior Digastric	0.050	0.978	digastric_ant_L	0.041	0.982
Geniohyoid	Geniohyoid	0.045	0.982	Geniohyoid_L	0.058	0.980
Mylohyoid_Post	Posterior Mylohyoid	0.058	0.978	Mylohyoid_Post_L	0.062	0.988
Mylohyoid_Ant	Anterior Mylohyoid	0.048	0.981	Mylohyoid_Ant_L	0.069	0.977
Stylohyoid_Lat	Lateral Stylohyoid	0.068	0.964	Stylohyoid_Lat_L	0.058	0.959
Stylohyoid_Med	Medial Stylohyoid	0.045	0.969	Stylohyoid_Med_L	0.033	0.974
Sterno_hyoid	Sternohyoid	0.045	0.973	Sterno_hyoid_L	0.051	0.988
SternoThyroid	Sternothyroid	0.042	0.976	SternoThyroid_L	0.049	0.988
Omo_hyoid	Omohyoid	0.068	0.982	Omo_hyoid_L	0.078	0.981
stern_mast	Sternocleidomastoid	0.062	0.980	stern_mast_L	0.056	0.990
cleid_mast	Cleidomastoid	0.031	0.989	cleid_mast_L	0.036	0.994
cleid_occ	Cleidooccipitalis	0.038	0.989	cleid_occ_L	0.049	0.987
scalenus_ant	Anterior Scalene	0.046	0.982	scalenus_ant_L	0.041	0.989
scalenus_med	Middle Scalene	0.062	0.982	scalenus_med_L	0.062	0.985
scalenus_post	Posterior Scalene	0.051	0.986	scalenus_post_L	0.054	0.985
long_cap_sklc4	Longus Capitis (skull-C4)	0.063	0.982	long_cap_sklc4_L	0.072	0.981
long_col_c1thx	Longus Colli (C1thorax)	0.044	0.985	long_col_c1thx_L	0.047	0.977
long_col_c1c5	Longus Colli (C1C5)	0.046	0.976	long_col_c1c5_L	0.065	0.980
long_col_c5thx	Longus Colli (C5thorax)	0.048	0.979	long_col_c5thx_L	0.063	0.955
trap_cl	Clavicular Trapezius	0.047	0.988	trap_cl_L	0.044	0.990
trap_acr	Acromial Trapezius	0.045	0.958	trap_acr_L	0.042	0.951
splen_cap_sklc6	Splenius Capitis (skull-C6)	0.051	0.982	splen_cap_sklc6_L	0.038	0.982
splen_cap_sklthx	Splenius Capitis (skullthorax)	0.051	0.962	splen_cap_sklthx_L	0.049	0.950
splen_cerv_c3thx	Splenius Cervicis (C3thorax)	0.041	0.980	splen_cerv_c3thx_L	0.040	0.985
semi_cap_sklc5	Semispinalis Capitis (skullC5)	0.029	0.985	semi_cap_sklc5_L	0.026	0.984
semi_cap_sklthx	Semispinalis Capitis (skullthorax)	0.042	0.980	semi_cap_sklthx_L	0.048	0.982
semi_cerv_c3thx	Semispinalis Cervicis (C3thorax)	0.057	0.979	semi_cerv_c3thx_L	0.049	0.982
levator_scap	Levator Scapulae	0.045	0.981	levator_scap_L	0.052	0.979
longissi_cap_sklc6	Longissimus Capitis (skullC6)	0.036	0.992	longissi_cap_sklc6_L	0.042	0.988
longissi_cerv_c4thx	Longissimus Cervicis (C4thorax)	0.042	0.985	longissi_cerv_c4thx_L	0.043	0.986
iliocost_cerv_c5rib	Iliocostalis Cervicis (C5rib)	0.048	0.986	iliocost_cerv_c5rib_L	0.058	0.988
rectcap_post_maj	Rectus Capitis Posterior Major	0.033	0.995	rectcap_post_maj_L	0.041	0.991
rectcap_post_min	Rectus Capitis Posterior Minor	0.043	0.958	rectcap_post_min_L	0.049	0.962
obl_cap_sup	Obliquus Capitis Superior	0.058	0.987	obl_cap_sup_L	0.048	0.984
obl_cap_inf	Obliquus Capitis Inferior	0.042	0.989	obl_cap_inf_L	0.039	0.991

Note: RMSE and R^2^ indicate the prediction error and goodness-of-fit between the neural network predictions and OpenSim results. Lower RMSE and R^2^ values closer to 1 reflect better prediction performance. For each muscle listed on the left, the corresponding entry on the right with suffix “_L” refers to the anatomically symmetric muscle on the left side of the body, sharing the same full name.

**Table 3:** Comparison of IoU values between patient and healthy subject head marker trajectories with paired *t*-test results.

Subject	TP IoU	RH IoU	LH IoU	Average IoU	*p*-Value
Patient	0.7325	0.7812	0.8024	0.7720	0.029 ^*^
Healthy Subject	0.9328	0.9221	0.9275	0.9275	

Note: IoU values for the three head markers (TP, RH, and LH) and the average IoU for each subject. Higher IoU values indicate greater similarity in the trajectory range. The *p*-value represents the result of the paired *t*-test for the comparison between the patient and healthy subject. * Statistically significant (p<0.05).

## Data Availability

The original data presented in the study are openly available in the ANSUR II dataset at https://www.openlab.psu.edu/ansur2/, accessed on 22 November 2025. The one million plausible anthropometric models generated in this study were created based on these data and are available from the corresponding author upon reasonable request.
